# Primary bile acid shapes peripheral immunity in inflammatory bowel disease-associated primary sclerosing cholangitis

**DOI:** 10.1042/CS20256078

**Published:** 2025-06-23

**Authors:** André A. Santos, David Pires, Vanda Marques, Nicole Alesina, Elisa Herraez, Pavel Roudnický, Pedro M. Rodrigues, Ana Godinho-Santos, Ana Catarina Bravo, Catarina Gouveia, Susana Saraiva, Luís Correia, Ricardo Crespo, João Pereira da Silva, Marília Cravo, David Potesil, Zbyněk Zdráhal, Jesus M. Banales, Jose J.G. Marin, Joana Torres, Cecília M.P. Rodrigues

**Affiliations:** 1 Research Institute for Medicines (iMed.ULisboa), Faculty of Pharmacy, Universidade de Lisboa, Lisboa, Portugal; 2 CIIS – Centro de Investigação Interdisciplinar em Saúde, Faculdade de Medicina, Universidade Católica Portuguesa, Lisboa, Portugal; 3 Experimental Hepatology and Drug Targeting (HEVEPHARM), Institute of Biomedical Research of Salamanca (IBSAL), University of Salamanca, Salamanca, Spain; 4 Center for the Study of Liver and Gastrointestinal Diseases (CIBERehd), Carlos III National Institute of Health, Madrid, Spain; 5 Mendel Centre of Plant Genomics and Proteomics, Central European Institute of Technology, Masaryk University, Brno, Czech Republic; 6 Department of Liver and Gastrointestinal Diseases, Biogipuzkoa Health Research Institute, Donostia University Hospital, University of the Basque Country (UPV/EHU), San Sebastian, Spain; 7 Ikerbasque, Basque Foundation for Science, Bilbao, Spain; 8 Division of Gastroenterology, Hospital Beatriz Ângelo, Loures, Portugal; 9 Division of Gastroenterology, ULS Santa Maria, Lisboa, Portugal; 10 Division of Gastroenterology, Hospital Lusíadas, Lisboa, Portugal; 11 Division of Gastroenterology, Hospital da Luz, Lisboa, Portugal; 12 Department of Biochemistry and Genetics, School of Sciences, University of Navarra, Pamplona, Spain

**Keywords:** bile acids, IBD, immune response, GCDCA, PSC

## Abstract

Primary sclerosing cholangitis (PSC) is a chronic cholestatic liver disease often associated with underlying inflammatory bowel disease (IBD). This study investigates how PSC predisposes individuals to altered inflammatory immune responses compared with IBD alone. A case–control study was conducted with a cohort of 75 patients, including 16 with PSC (14 with concomitant IBD), 39 with IBD alone, and 20 controls. Serum bile acid profile, proteomic analysis, and immune-related gene expression in the colon tissue were examined. Colonic tissue from PSC patients exhibited up-regulation of immune regulation and inflammatory signaling mRNA markers, including *LGR5, IL-8, CCL2, COX2, TWIST1,* and *SNAIL*. Additionally, PSC patients displayed a distinct proinflammatory serum proteomic signature and moderate elevation of some bile acids, such as glycochenodeoxycholic acid (GCDCA). Co-incubation of human-derived monocytes with GCDCA partially replicated the inflammatory profile observed in PSC. These findings suggest that circulating bile acids modulate the peripheral immune system proinflammatory response, contributing to the unique PSC phenotype.

## Introduction

Primary sclerosing cholangitis (PSC) is a chronic, progressive cholestatic liver disease of unknown etiology, characterized by inflammation, fibrosis, and strictures affecting the intrahepatic and extrahepatic bile ducts [1]. Despite being a rare disease, PSC remains the fifth most common indication for liver transplant in the USA and bears a dismal prognosis [2,3]. In PSC, an abnormally high amount of bile acids is accumulated in the bloodstream due to bile duct obstruction, hepatocyte injury, and impaired bile flow [4]. Inflammation and scarring of the bile ducts disrupt bile acid secretion into the gut, while damaged hepatocytes and altered bile acid transporter activity exacerbate leakage into the circulation [5]. These mechanisms lead to elevated bile acid levels in the blood, driving systemic inflammation and immune dysregulation.

Traditionally known for their role in digestion, bile acids are now recognized as key signaling molecules that modulate immune responses. Dysregulated bile acids metabolism contributes to liver inflammation and PSC progression, particularly through the toxic effects of unconjugated bile acids [6]. Understanding whether circulating bile acids are involved in PSC pathogenesis is crucial for uncovering new therapeutic targets. However, the influence of the peripheral immune system response in PSC in the context of inflammation remains unexplored.

PSC often co-exists with inflammatory bowel disease (IBD), affecting up to 80% of PSC patients [3]. IBD, including Crohn’s disease (CD) and ulcerative colitis (UC), involves chronic inflammation of the gastrointestinal tract, with the gut microbiota playing a crucial role [7]. PSC-associated IBD presents a distinct phenotype, most frequently characterized by pancolitis, rectal sparing, and a significantly increased risk of colorectal neoplasia, more commonly located in the right colon. Interestingly, inflammation and colitis-associated neoplasia are typically more prominent in the right colon of PSC-IBD patients, compared with those with IBD alone [8]. Emerging evidence suggests that dysbiosis, altered intestinal permeability, and aberrant T cell homing contribute to both diseases, and this specific phenotype [9-12]. Nonetheless, the underlying mechanisms driving the increased risk for IBD in PSC patients and its distinct phenotype remain poorly understood.

Given the complexity of PSC, this study integrates serum bile acid profiling, proteomics, tissue gene expression, and *in vitro* analysis to investigate how circulating bile acids modulate the peripheral immune system and contribute to gut inflammation in PSC patients.

## Methods

### Patient enrollment and ethics

This case–control study included patients with PSC, IBD alone, and control individuals undergoing colonoscopy for colorectal cancer screening or polyp surveillance. Inclusion criteria included age greater than or equal to 18 years, patient willing and able to sign the informed consent, confirmed diagnosis of PSC by conventional criteria [1], and confirmed diagnosis of IBD and PSC. Sample collection was only performed after patient informed and written consent, according to World Medical Association (WMA) Declaration of Helsinki, Ethical Principles for Medical Research Involving Human Subjects, as well as under the Portuguese law DL 43/04 de 19 de Agosto, DR I Série, and after approval by the Ethical Committee (Ref. 0028/2014_RMEB). Demographic and clinical data were collected. Patients with PSC were asked to stop ursodeoxycholic acid treatment two weeks before tissue collection to avoid interfering with the bile acid profile. During colonoscopy, a biopsy from the right colon was collected and preserved in RNAlater solution (Thermo Fisher Scientific). Serum samples were collected on the same day the patient underwent colonoscopy and preserved at −80°C.

### Serum bile acid extraction and analysis

Bile acid-related standards were obtained from Sigma-Aldrich (Merck, Madrid, Spain), except 7α-hydroxy-4-cholesten-3-one (C4) and nor-deoxycholic acid (nor-DCA), which were acquired from Avanti Polar Lipids (Alabaster) and Toronto Research Chemicals (North York), respectively. C4 concentration was measured after acetonitrile precipitation/extraction [13] of 400 ml of serum by high-performance liquid chromatography–tandem mass spectrometry (HPLC-MS/MS) as previously described [14]. Bile acid profiling in serum was carried out using a modification [15] of a previously described method [16], on a 6420 Triple Quad liquid chromatography (LC)-MS device (Agilent Technologies). Chromatographic separation was performed with gradient elution using a Zorbax Eclipse XDB-C18 column (150 mm×4.6 mm, 5 µm), kept at 35°C, and a flow rate of 0.5 ml/min. The initial mobile phase was 73:27 methanol/water, both containing 5 mM ammonium acetate and 0.01% formic acid, pH 4.6, and it was modified to 97:3 methanol/water over 10 min, and then returned to 73:27 in 1 min. Electrospray ionization in negative mode was performed under the following conditions: gas temperature 350°C, gas flow 11 l/min, nebulizer 45 psi, capillary voltage 2500 V. MS/MS acquisition was achieved in multiple reaction monitoring mode using the specific m/z transitions: [M-H]- ion to 80.2 for taurine-conjugated bile acids, and [M-H]- ion to 74 for glycine-conjugated bile acids. Since unconjugated bile acids do not generate characteristic ion fragments, transitions from unfragmented precursor molecular ions 375.3 to 375.3, 391.3 to 391.3, and 407.1 to 407.1 were selected for non-amidated monohydroxylated, dihydroxylated, and trihydroxylated free bile acids, respectively. Transition 377.0 to 331.3 m/z was followed for the internal standard nor-DCA.

### Primary cell isolation and culture conditions

Human monocytes were obtained by isolating CD14^+^ monocytes from buffy coats of healthy blood donors provided by the national blood institute (Instituto Português do Sangue e da Transplantação, Lisbon, Portugal) using the MojoSort magnetic cell separation system (Biolegend) and following a previously described procedure with minor alterations [17]. Briefly, peripheral blood mononuclear cells were first isolated by density gradient centrifugation with Ficoll-Paque Plus (Cytiva), and then treated with Human TruStain FcX (Biolegend) and incubated with CD14-specific magnetic beads (Biolegend). CD14^+^ monocytes bound to the beads were magnetically recovered and cultivated in Roswell Park Memorial Institute (RPMI)-1640 medium with 10% (v/v) fetal bovine serum, 1 mM sodium pyruvate, 10 mM HEPES, and 0.1% β-mercaptoethanol (all from Gibco). To induce monocyte differentiation, 20 ng/ml recombinant human M-CSF (Thermo Fisher Scientific) was added to the medium to obtain macrophages. To determine the impact of glycochenodeoxycholic acid (GCDCA) in monocyte differentiation, cells were treated with 50 µM GCDCA on days 0, 3, and 6 post-isolation. Fresh cell culture medium was added every 3 days until day 6 post-isolation. On day 6, cells were stimulated with 100 ng/ml lipopolysaccharides (LPS) (Merck) or 20 ng/ml recombinant human tumor necrosis factor alpha (TNF-α) (Biolegend). Following 24 h of stimulation, the cells were ready for subsequent assays.

### Flow cytometry

Human monocyte-derived macrophages were seeded in 24-well plates at a density of 3 × 10^5^ cells per well and differentiated as previously described. Cells were recovered with 5 mM EDTA/PBS solution on the analysis day. Recovered cells were treated with Human TruStain FcX (Biolegend) and stained for 30 min with antibodies (Biolegend). The antibodies selected constitute receptors of microbial molecules and markers of macrophage polarization. CD14 (clone M5E2, #301839) is a monocyte/macrophage marker and an LPS receptor; CD16 (clone 3G8, #302067) is a marker for non-classical monocytes; CD80 (clone W17149D, #375407) is a co-stimulator molecule for T-lymphocyte activation and an M1-macrophage marker; CD163 (clone GHI/61, # 333629) is a monocyte/macrophage marker particularly rich in tumorigenic environments; CD204 (clone 7C9C20, #371903) is a macrophage receptor associated with lipid metabolism, inflammatory disorders, liver disease, and cancer; CD206 (clone 15–2, #321116) is a receptor for microbial glycans and glycoproteins and a marker of M2-macrophages; and human leukocyte antigen (HLA-DR) (clone L243, #307673) is a class II HLA molecule of antigen-presenting cells. Antibodies specific for other immune cells were used as controls to exclude lymphocytes and dendritic cells. Following immunostaining, the cells were fixed with 4% paraformaldehyde for 15 min. Samples were analyzed in the Cytek Aurora full-spectrum cytometer (Cytek Biosciences), and data analysis was performed in FCS Express™ (De Novo Software). Serum samples were collected, and levels of interferon-gamma (IFN-γ), interleukin-6 (IL-6), IL-8, IL-10, IL-17A, and interleukin-33 (IL-33) were quantified with the LEGENDplex^TM^ Multi-Analyte Flow Assay kit (LEGENDplex^TM^ Human Inflammation Panel 1 Mix and Match, BioLegend), according to the manufacturer’s instructions. Cytokine concentrations were determined using the LEGENDplex data analysis software (BioLegend).

### Patient fecal sample collection for calprotectin analysis

Collection of fecal samples was performed by the patients immediately after evacuation using the EasySampler^®^ Stool Collection Kit (ALPCO Diagnostics). The samples were always collected before colonoscopy to avoid the effects of bowel preparation. Samples were preserved at 4°C for a maximum of 24 h before preservation at −80°C. Calprotectin analysis was performed using Quantum Blue^®^ Calprotectin extended (Buhlmann) together with Calex cap extraction tubes (Buhlmann), according to the manufacturer’s instructions.

### Proteomic analysis of serum, sample preparation, and analysis

Serum samples underwent depletion of highly abundant protein using High Select™ Top14 Abundant Protein Depletion Mini Spin Columns (Thermo Fisher Scientific). The depleted serum samples were used for filter-aided sample preparation (FASP, 30 kDa cut-off cartridges) as described elsewhere using 0.5 μg of trypsin (sequencing grade; Promega) [18]. The resulting peptides were extracted into LC-MS vials with 2.5% formic acid in 50% acetonitrile and 100% acetonitrile with the addition of polyethylene glycol (20,000; final concentration 0.001%, [19]) and concentrated in a SpeedVac concentrator (Thermo Fisher Scientific); these peptides were taken for LC-MS analysis. LC-MS/MS analyses of all peptide mixtures were done using the Ultimate 3000 RSLCnano system connected to Orbitrap Exploris 480 mass spectrometer (Thermo Fisher Scientific). Prior to LC separation, tryptic digests were online concentrated and desalted using a trapping column (300 μm×5 mm, μPrecolumn, 5 μm particles, Acclaim PepMap100 C18, Thermo Fisher Scientific; temperature of 40°C). After washing the trapping column with 0.1% formic acid, the peptides were eluted (flow rate – 250 nl/min) from the trapping column onto an analytical column (EASY spray column, Acclaim Pepmap100 C18, 2 µm particles, 75 μm×250 mm; Thermo Fisher Scientific) by 90-min linear gradient program (5–37% of mobile phase B; mobile phase A: 0.1% formic acid in water; mobile phase B: 0.1% formic acid in 80% acetonitrile). Equilibration of the trapping column and the analytical column was done prior to sample injection into the sample loop. The analytical column was installed in the EASY-Spray ion source (Thermo Fisher Scientific) according to the manufacturer’s instructions with the column temperature of 40°C. Spray voltage and sweep gas were set to 1.9 kV and 1 (Arb), respectively. Data were acquired in a data-independent acquisition mode (DIA). The survey scan covered the m/z range of 350–1400 at a resolution of 60,000 (at m/z 200) and a maximum injection time of 55 ms. HCD MS/MS (27% relative fragmentation energy) was acquired in the range of m/z 200–2000 at 30,000 resolution (maximum injection time 55 ms). Overlapping windows scheme in the m/z range from 400 to 800 was used as isolation window placements. A detailed transitions list is available upon request. DIA data were processed in DIA-NN3 (version 1.8 [20]) in library-free mode against modified cRAP database (based on http://Control.thegpm.org/crap/; 111 sequences in total) and UniProtKB protein database for Homo sapiens (https://ftp.uniprot.org/pub/databases/uniprot/current_release/knowledgebase/reference_proteomes/Eukaryota/UP000005640/UP000005640_9606.fasta.gz; version 2022/12, number of protein sequences: 20,594). No optional, carbamidomethylation as fixed modification and trypsin/P enzyme with 1 allowed missed cleavages and peptide length 7–30 were set during the library preparation. False discovery rate (FDR) control was set to 1% FDR. Mass spectrometry (MS)1 and MS2 data accuracies, as well as scan window parameters, were set based on the initial test searches (median value from all samples ascertained parameter values). Match-between-runs (MBR) was switched on. Protein MaxLFQ intensities reported in the DIA-NN main report file were further processed using the software container environment (https://github.com/OmicsWorkflows/KNIME_docker_vnc), version 4.6.3 a. Processing workflow is available upon request. Briefly, it covered: a) removal of low-quality precursors and contaminant protein groups, b) protein group intensities log_2_ transformation and normalization, c) filtering out of protein groups not quantified in at least three replicates of single sample type prior to imputation of missing values and differential expression analysis using the LIMMA statistical test with *P* values adjustment for multiple comparisons done using the Benjamini-Hochberg technique. Proteins were considered as statistically changing when the adjusted *P* value was lower than 0.05 and the absolute value of log2 fold change (|log2FC|) was higher than 1. MS proteomics data were deposited to the ProteomeXchange Consortium via the PRIDE [21] partner repository under dataset identifier PXD005011. STRING analysis [22] for the proteins with statistically higher intensity (adjusted *P* value <0.05, log2FC ≥1) observed in the IBD alone versus PSC group was performed using the website https://string-db.org/ version 12.0. Non-connected proteins were removed from the final figure. Analysis resume is available upon request.

### Gene expression analysis

Total RNA was extracted from right colon tissue using Trizol Reagent (Thermo Fisher Scientific) and converted into cDNA using NZY Reverse Transcriptase (NZYTech), all according to the manufacturer’s instructions. qPCR was performed in a QuantStudio™ 7 lex Real-Time PCR System (Thermo Fisher Scientific). Primer sequences are listed in Supplementary Table S1. Two independent reactions for each primer set were performed in a total volume of 12.5 μl containing 2 x sensiFAST SYBR Hi-ROX kit (Bioline) and 0.6 μM of each primer (Stabvida). The relative amounts of each gene were calculated based on 2^-ΔΔCt^ method normalized to the level of β-actin and expressed as fold change from the control group.

### Statistical analysis

Statistical analysis was performed with GraphPad Prism version 9 software (La Jolla). The ROUT (Q=1%) method was used to identify outliers. D'Agostino & Pearson test normality and Lognormality test were used to evaluate sample distribution for each analysis performed. According to data normality distribution, differences between two groups were assessed with the t-test or Mann–Whitney U test. Differences between three or more groups were evaluated using Kruskal–Wallis or one-way analysis of variance (ANOVA), followed by Dunn’s or Tukey multiple comparison tests, respectively. For categorical data, a chi-squared test was used. A *P* value <0.05 was considered statistically significant. Results were presented as mean ± standard error of the mean (SEM).

## Results

### Patient characterization

A total of 75 individuals were included, 16 patients with PSC (14 with concomitant IBD), 39 patients with IBD, and 20 controls, as described in Table 1. The control group primarily includes individuals undergoing routine colorectal cancer (CRC) screening, which generally involves an older population. In contrast, patients with PSC-IBD and IBD alone are often diagnosed at a younger age. Additionally, the observed higher proportion of females in the control group likely reflects demographic patterns in screening participation, where women may be more likely to engage in preventive healthcare. This may partly explain the observed trends in age and sex distribution. Nevertheless, no statistically significant differences were found between the groups in terms of age or sex.

**Table 1:** Clinical characteristics of patients included in PSC-IBD, IBD, and control groups.

Characteristics	PSC (*n*=16)	IBD (*n*=39)	Control (*n*=20)	*P-value*
Age (median, IQR)	42 (38–47)	49 (36–62)	62 (42–67)	0.131
Male sex, *n* (%)	9 (56)	18 (46)	7 (35)	0.440
IBD, *n* (%)				
CD	5 (31)	18 (46)		0.629
UC	8 (50)	21 (54)	NA	
Unclassified	1 (6)	NA		NA
No IBD detected	2 (13)	NA		NA
Calprotectin (median, IQR)	321 (99–746)	217 (78–935)	45 (39–205)	0.016
Disease duration (PSC), y (median, IQR)	9 (5–12)	NA	NA	NA
Disease duration (IBD), y (median, IQR)	14 (9–17)	9 (5–20)	NA	0.852
Treatment, *n* (%)				
5-ASA	9 (56)	18 (46)		0.496
Steroids	2 (13)	1 (3)	NA	0.141
IMM (Tracrolimus, MMF, AZA, or MTX)	3 (19)	13 (33)		0.280
Biologic (IFX, ADA, or UST)	2 (13)	14 (36)		0.083
Endoscopic inflammation at colonoscopy, *n* (%)				
Terminal ileum	4 (25)	4 (10)	NA	0.159
Right colon	6 (38)	12 (31)		0.629
Left colon	3 (19)	11 (28)		0.465
UDCA, *n* (%)				
Permanent	7 (44)			
Stopped 15 days before colonoscopy	7 (44)	NA	NA	NA
No treatment	2 (13)			
Cirrhosis, *n* (%)	3 (19)	NA	NA	NA
History of liver transplant, *n* (%)	2 (13)	NA	NA	NA

5-ASA, 5-aminosalicylic acidCD, Crohn’s diseaseIBD, inflammatory bowel disease;IMM, immunomodulator;MMF, mycophenolate mofetil;AZA, azathioprine;MTX, methotrexate;IFX, infliximab;ADA, adalimumab;UST, ustekinumab;IQR, interquartile range;PSC, primary sclerosing cholangitis;UC, ulcerative colitis;UDCA, ursodeoxycholic acid.

Among the PSC group, two patients had PSC alone (confirmed by colonoscopy and biopsies showing no IBD), eight had UC, five had CD, and one had an undetermined form of IBD. Three of the PSC patients had cirrhosis at the time of recruitment, and two were subjected to liver transplant due to recurrent cholangitis. Active disease was documented in four PSC-IBD patients and 11 IBD-alone patients. No colorectal neoplasia was detected in either patients or controls. The median duration of IBD was 14 years interquartile range (IQR 9–17) for PSC patients and 9 years (IQR 5–20) for IBD-only patients. PSC-IBD patients were receiving treatment with 5-aminosalicylic acid (5-ASA) (56%), steroids (13%), immunomodulators (19%), or biologics (13%). In the IBD group, 21 patients had UC and 18 had CD, with treatment consisting of 5-ASA (46%), steroids (3%), immunomodulators (33%), and biologics (36%). Fecal calprotectin levels were significantly elevated in both PSC patients (median 321 ng/μl, IQR 99–746) and IBD patients (median 217 ng/μl, IQR 78–935) compared with controls (*P=*0.484 for PSC, *P=*0.0295 for IBD). However, no significant differences were observed between the PSC and IBD groups.

### Serum conjugated primary bile acids are increased in PSC patients

**Figure 1:**
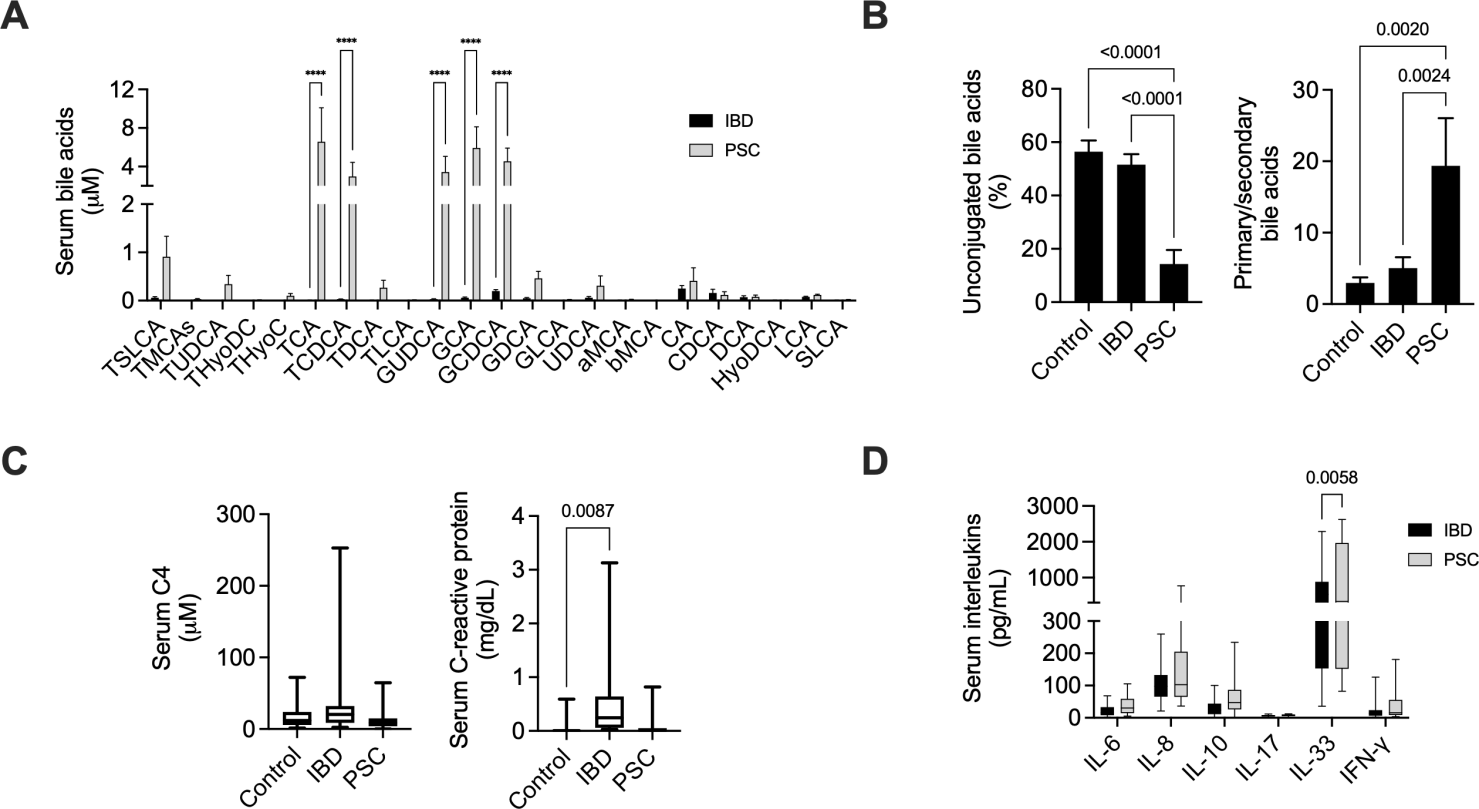
Patients with PSC have increased conjugated primary bile acids and inflammatory factors when compared with IBD alone. **(A**) Serum total bile acid profile. (**B**) Percentage of unconjugated bile acids and ratio between primary and secondary bile acids in serum. (**C**) Serum 7α-hydroxy-4-cholesten-3-one (**C4**) and C-reactive protein quantification. (**D**) Serum levels of IL-6, IL-8, IL-10, IL-17, IL-33, and IFN-γ. Results are expressed in fold change as mean values with error bars ± SEM.

Although not extremely high, total bile acids serum levels were significantly more elevated in patients with PSC when compared with those with IBD. This was particularly so for conjugated primary bile acids such as taurocholic acid (TCA), taurochenodeoxycholic acid, glycocholic acid, and GCDCA, as well as secondary BA glycoursodeoxycholic acid (all *P*<0.0001; Figure 1A). BA analysis showed decreased proportions of unconjugated-to-amidated BAs in PSC patients compared with both IBD alone and controls (both *P*<0.0001), and with increased ratios of primary-to-secondary BAs (*P*=0.0024 and *P*=0.0020) (Figure 1B). Serum levels of the BA precursor C4 (*P*=0.0543), commonly used as an indirect marker of BA synthesis rate by the liver, and C-reactive protein (*P*=0.0950) tended to decrease in PSC patients compared with IBD alone patients (Figure 1C). Moreover, serum levels of IL-33 were significantly increased in PSC patients (*P*<0.0058; Figure 1D).

### Patients with PSC present a more pro-inflammatory serum proteomic profile

**Figure 2:**
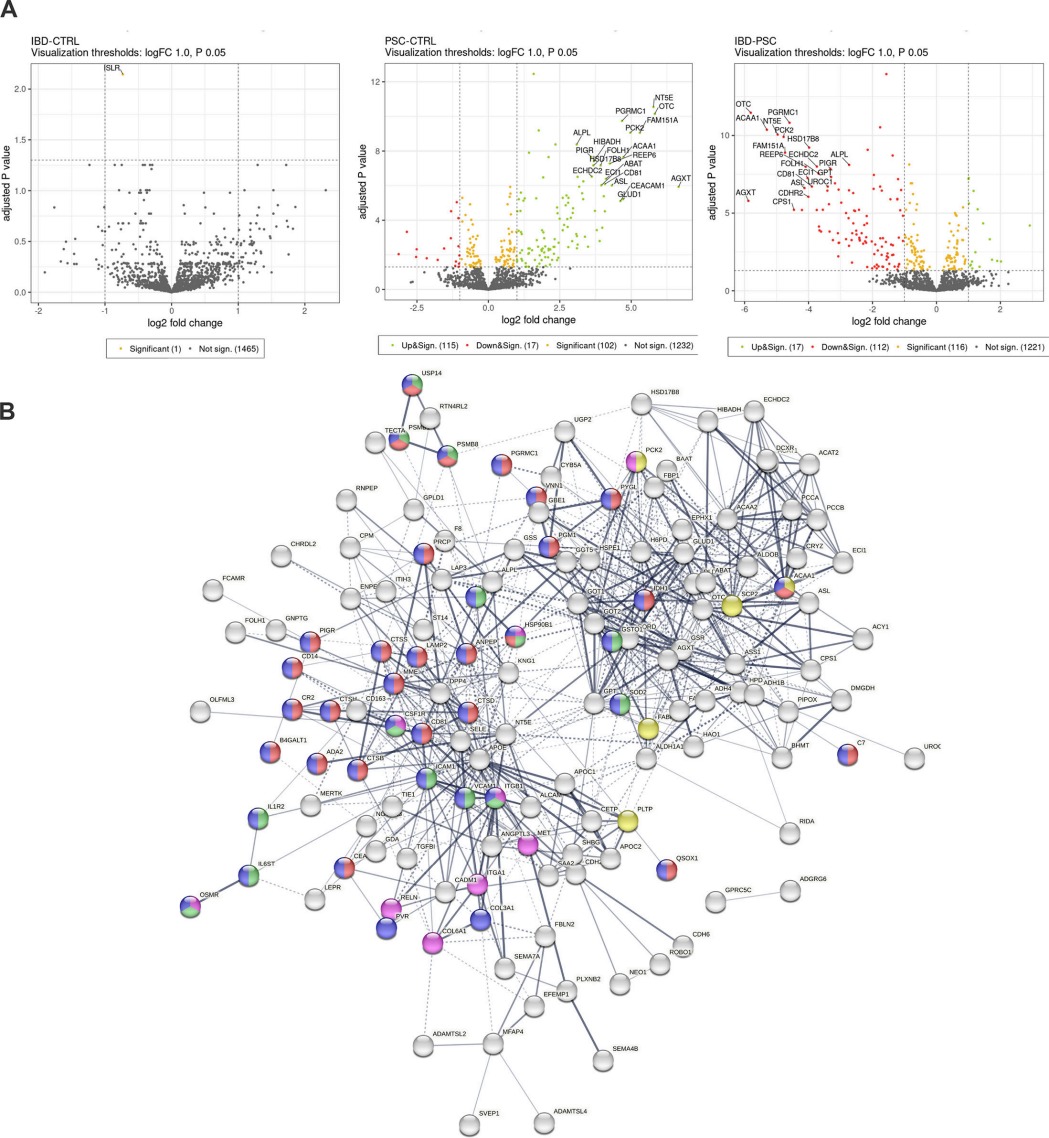
Serum proteomic analysis reveals an increase in peripheral pro-inflammatory markers in PSC patients. **(A**) Serum proteomic analysis after albumin depletion of IBD vs. control, PSC vs. control, and IBD vs. PSC. (**B**) String analysis of the 112 significant proteins increased in PSC serum samples; orange – immune receptor activity; yellow – PPAR signaling pathway; magenta – PI3K-Akt signaling pathway; light green – cell–cell adhesion via plasma-membrane adhesion molecules; maroon – acute inflammatory response; purple – vasodilation; pink – inflammatory response; gray – leukocyte cell–cell adhesion; light blue – immune system process; black – acute-phase response; dark green – neutrophil degranulation; red – innate immune system; blue – immune system; lime green – signaling by Interleukins; cyan – immunoregulatory interactions between a lymphoid and a non-lymphoid cell. Proteomic data are log_2_ fold change and log_10_ adjusted *P* values.

An in-depth serum proteomic analysis revealed no statistically significant differences between the controls and IBD (Figure 2A). However, serum samples of patients with PSC showed notable changes compared with controls, with 102 differently expressed proteins (adjusted *P* value <0.05 and |log2FC| > 1). Direct comparison between patients with PSC and IBD revealed 116 differently expressed proteins, with 112 proteins of them up-regulated in PSC. STRING analysis revealed 683 edges, with an average node degree of 8.54 and an average local clustering coefficient of 0.445, contributing to PPI enrichment *P-value* < 1.0e-16. Notably, pathways related to immune modulation were significantly altered, namely immune receptor activity, the PI3K-AKT signaling pathway, cell–cell adhesion via plasma membrane adhesion molecules, acute inflammatory response, and innate immune system response among others (Figure 2B, Supplementary Table S2).

### GCDCA modulates peripheral immune differentiation toward a pro-inflammatory phenotype

**Figure 3:**
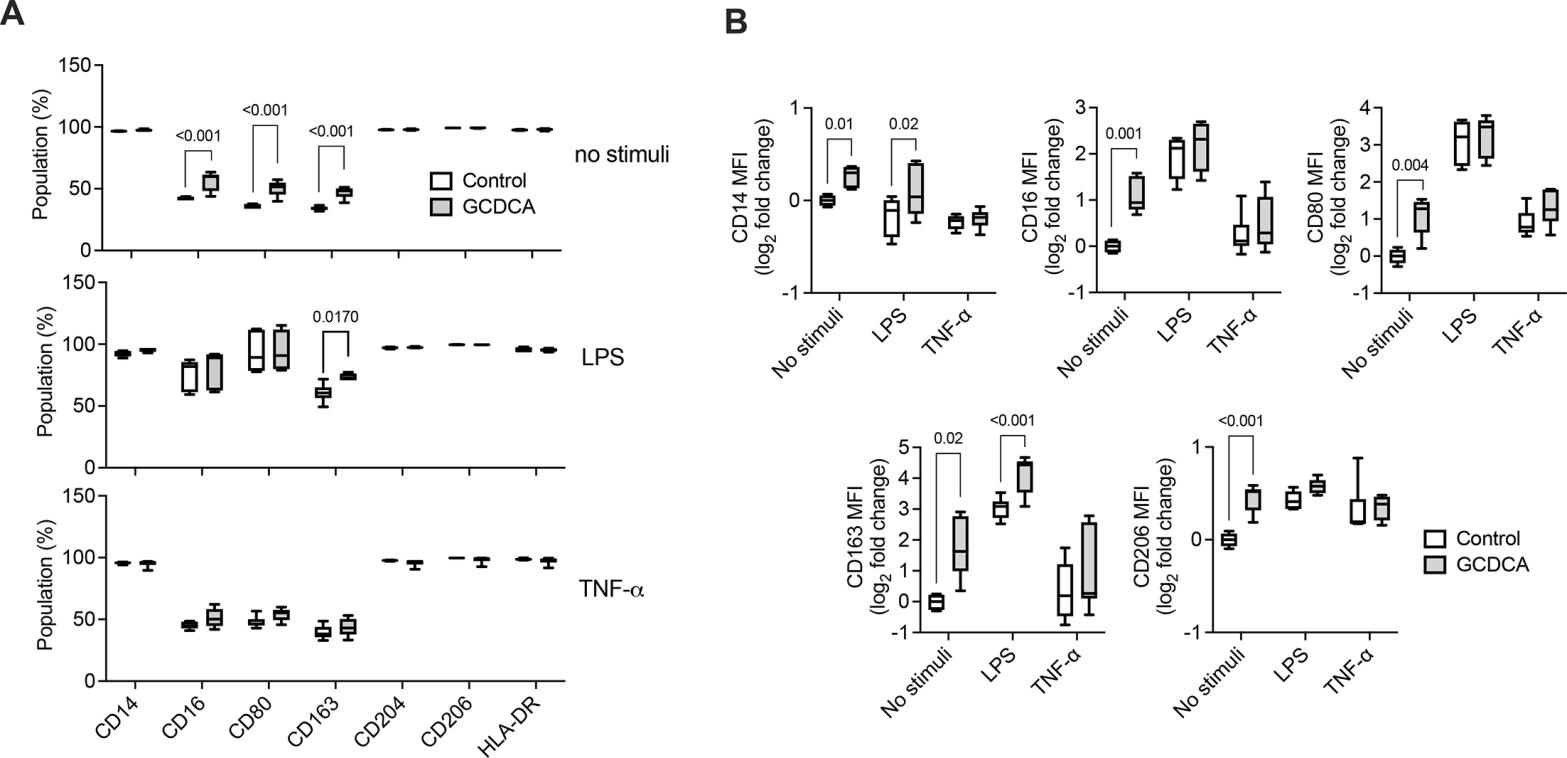
Effect of GCDCA on monocytes *in vitro*. **(A**) Percentage of differentiated monocytes expressing CD14, CD16, CD80, CD163, CD204, CD206, and HLA-DR, differentiated with 50 µM of GCDCA and exposed to LPS (100 ng/ml) or recombinant human TNF-α (20 ng/ml) for 24 h. (**B**) Mean fluorescence intensity (MFI) of differentiated monocytes expressing CD16, CD14, CD80, CD206, and CD163, differentiated with 50 µM of GCDCA and exposed to LPS (100 ng/ml) or recombinant human TNF-α (20 ng/ml) for 24 h. Results are expressed in fold change as mean values with error bars ± SEM.

Given the increased serum levels of some BAs, like GCDCA, and the pro-inflammatory peripheral immune profile in patients with PSC, *in vitro* studies were performed using primary monocytes isolated from human blood. When GCDCA was used concomitantly with M-CSF for monocyte differentiation, it altered the differentiated monocyte response. Specifically, GCDCA increased the proportion of CD16^+^, CD80^+^, and CD163^+^ cells (*P*<0.0001; Figure 3A and B). To further elucidate this modulatory effect, LPS and TNF-α were used to stimulate the differentiated monocytes, inducing an inflammatory response. Interestingly, in LPS-stimulated monocytes, GCDCA treatment further enhanced the CD163^+^ population (*P*<0.0001), underscoring its potential role in promoting a pro-inflammatory phenotype

### Key markers highlight cancer susceptibility in the colon of PSC patients

**Figure 4:**
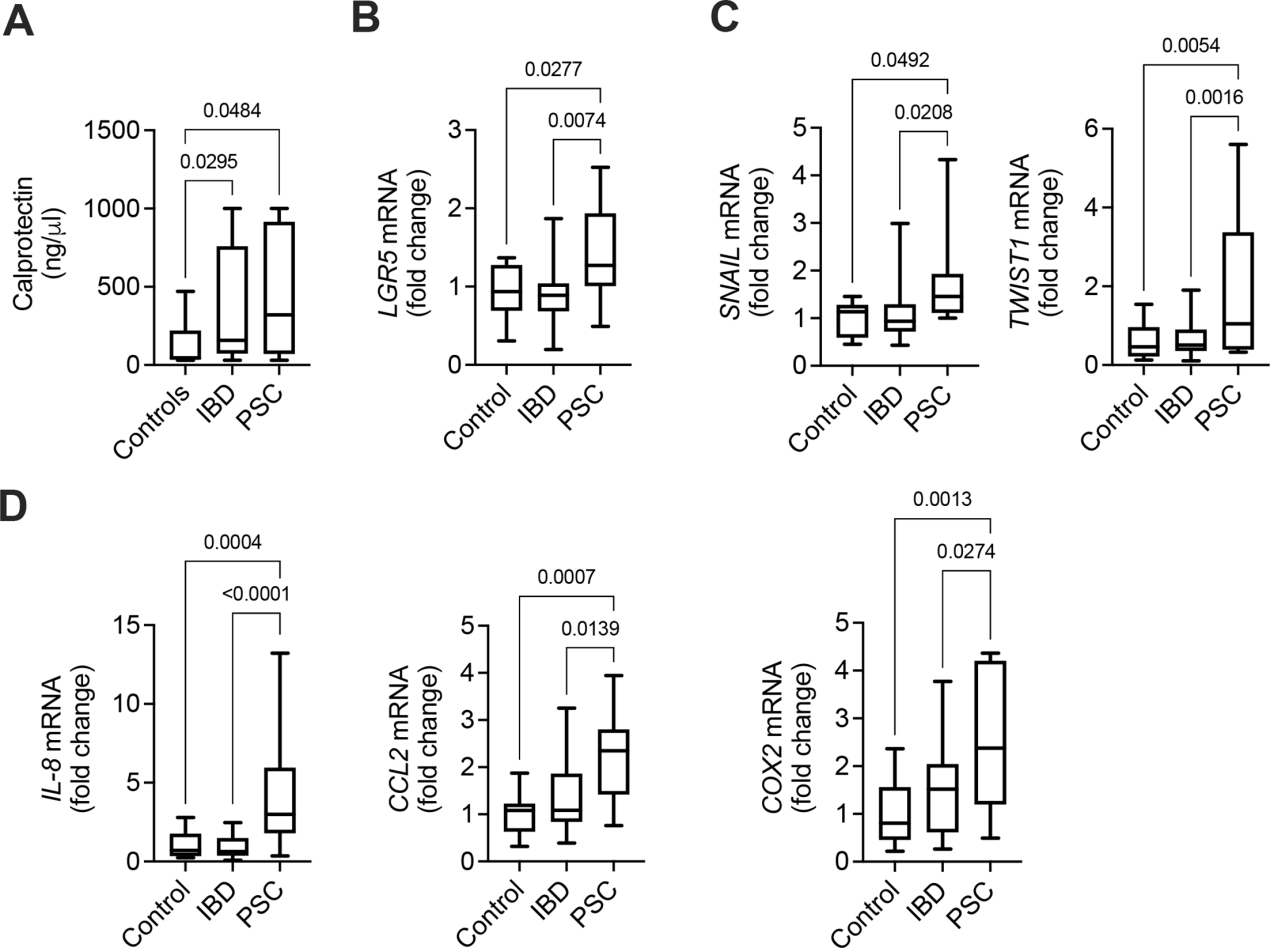
Altered colon immune system response in PSC patients. (**A**) Fecal calprotectin quantification. (**B**) Right colon mRNA expression of intestinal stem cell and regeneration mRNA marker *LGR5*. (**C**) Right colon mRNA expression of intestinal epithelial–mesenchymal transition markers *SNAIL* and *TWIST1.* (**D**) Right colon mRNA expression of pro-inflammatory tumor microenvironment markers *IL-8*, *CCL2*, and *COX2*. qPCR results are expressed in fold change as mean values with error bars ± SEM. Proteomic data are log_2_ fold change and log_10_ adjusted *P* values.

Considering the distinct peripheral alterations observed in PSC patients, fecal calprotectin levels were assessed to evaluate inflammatory differences. However, no significant differences were found between PSC and IBD groups (Figure 4A). Given the absence of differences in generalized inflammation, we focused on markers associated with tumor progression and microenvironment regulation. These included *LGR5* as a stem cell and regeneration mRNA marker, *SNAIL* and *TWIST1* as markers of epithelial–mesenchymal transition; and *IL-8*, *CCL2*, and *COX2* as pro-inflammatory tumor microenvironment markers.

Assessment of colonic epithelial homeostasis showed a significant increase in LGR5 expression in PSC patients compared with the IBD and control groups (*P*=0.0074 and *P*=0.0277, respectively; Figure 4B). Evaluation of epithelial–mesenchymal transition markers showed increased expression of *SNAIL* (*P*=0.0208 and *P*=0.0492) and *TWIST1* (*P*=0.0016 and *P*=0.0054) in PSC compared with IBD and controls (Figure 4C). Similarly, analysis of pro-inflammatory tumor microenvironment markers demonstrated elevated expression of *IL-8* (*P*<0.0001 and *P*=0.0003), *CCL2* (*P*=0.0139 and *P*=0.0007), and *COX2* (*P*=0.0274 and *P*=0.0013) in the right colon of PSC patients compared with both IBD alone and control groups (Figure 4D).

## Discussion

In this study, we identified a distinct peripheral immune phenotype in patients with PSC, a chronic cholestatic liver disease frequently associated with underlying IBD. Our findings highlight a compelling link between circulating bile acids and immune modulation, as GCDCA was shown to promote monocyte differentiation into CD163^+^ tumor-associated macrophages, which exhibit pro-tumorigenic properties. These effects were further supported by the up-regulation of tumor progression markers, including *LGR5*, *TWIST1*, and *COX2*, in PSC-IBD colon tissue. This suggests that bile acid-mediated immune dysregulation may contribute to the development of a tumor-promoting microenvironment. Together, these findings strengthen the hypothesis that PSC-associated systemic inflammation and bile acid dysregulation drive immune cell recruitment and functional reprogramming, thus perpetuating colon-specific inflammation and tumorigenic processes in PSC-IBD.

PSC is characterized by reduced bile acid secretion from the liver into the gut and increased release to the systemic circulation. In this study, we identified GCDCA as one of the most prevalent bile acids in serum. GCDCA is a conjugated primary bile acid and is considered the main toxic component of bile and serum of patients with cholestasis [23]. Importantly, this and other bile acids have been shown to act as co-carcinogenic agents when accumulated in the liver [24]. Studies in rats have demonstrated that GCDCA-induced hepatocyte apoptosis is mediated through early regulation of the intracellular protein kinase C signaling pathway, which plays a critical role in regulating key liver functions [25]. Collectively, these findings highlight the multifaceted toxicity of GCDCA and its pivotal role in cholestasis-associated hepatocyte injury.

Interestingly, an exploration of the serum proteomic profiles in patients with PSC revealed significant alterations in pathways related to immune regulation and inflammatory signaling. Notably, GCDCA was found to modulate monocyte differentiation *in vitro*, promoting an increase in CD16, CD80, and CD163, similar to the response observed during LPS stimulation. These markers are strongly associated with monocyte origin and macrophage polarization [26,27]. Among these, CD163 exhibited the most pronounced response to GCDCA treatment, even in the presence of LPS. CD163 is a hallmark of tumor-associated macrophages, which are known to support tumor initiation, progression, and metastasis through their immunosuppressive and pro-angiogenic properties [28-30]. Curiously, in patients with PSC, serum GCDCA levels positively correlated with elevated levels of serum CSF1R and CD163. Furthermore, our *in vitro* experiments demonstrated that GCDCA-conditioned monocytes exhibited phenotypic and molecular features consistent with those observed in the serum of PSC patients, including the up-regulation of markers such as CD163. This partial recapitulation of the PSC serum profile suggests that GCDCA, a liver-derived primary bile acid, exerts immunomodulatory effects beyond the liver by directly influencing monocyte differentiation and polarization. These altered monocytes likely contribute to the systemic inflammatory environment characteristic of PSC and may adopt distinct functions upon recruitment to inflamed tissues, thereby linking bile acid dysregulation to peripheral immune activation and offering novel insight into the pathogenesis of PSC-related inflammation.

PSC-related IBD has long been considered a distinct and unique colon inflammatory phenotype [8,31-34]. However, the precise mechanisms underlying this phenomenon remain poorly understood. Calprotectin is a valuable noninvasive marker of intestinal inflammation; however, PSC-IBD is known to present with distinct clinical and histological features compared with conventional IBD, including right-sided predominance and less symptomatic disease [35]. These factors may contribute to the absence of significant differences in calprotectin levels between PSC-IBD and IBD patients. Despite the non-significant trend toward higher calprotectin levels in the PSC-IBD group, we acknowledge that this may reflect subtle differences in the inflammatory milieu or disease activity not captured by conventional scoring. While we anticipate potential differences due to the unique characteristics of PSC-IBD [36], the small cohort and inter-individual variability likely limited our ability to detect statistically significant differences. Through gene expression analyses of colon tissue, we identified significant increases in markers of tumor progression and microenvironment regulation, including *LGR5*, *TWIST1*, *SNAIL*, *IL-8*, *CCL2*, and *COX2*, in the colon of PSC patients compared with those with IBD alone. The apparent discordance between calprotectin and macrophage modulation suggests that while active inflammation is present in both groups (as indicated by high calprotectin), the underlying tissue environment in PSC-IBD may be more skewed toward a chronic, macrophage-rich, and pro-oncogenic state. Rather than indicating a reduced inflammatory response, the differential expression of these markers may point to a qualitatively distinct inflammatory microenvironment, potentially more localized and associated with long-term epithelial changes and oncogenic signaling pathways. The distinct immune and inflammatory profile observed in PSC patients, driven in part by bile acid-mediated immune dysregulation and chronic inflammatory signaling, appears to contribute to a colon tumor-promoting microenvironment. Interestingly, GCDCA has been shown to trigger the expression of inflammatory chemoattractant *IL-8* and *CCL2* in hepatocytes [37], and it has been positively correlated with colorectal cancer development and progression [38]. Accordingly, increased expression of *IL-8* and *CCL2* was observed in the colon tissue of patients with PSC. In line with the altered cytokine profile, increased IL-33, known as a chromatin-associated cytokine known to influence epigenetics [39], has been correlated with the development of several types of cancer, including progression to cholangiocarcinoma [40,41]. This is further supported by the up-regulation of serum markers such as CD163, associated with tumor-associated macrophages, alongside the elevated expression of tumor progression and microenvironment regulation markers in colon tissue. Even though several PSC patients receive immunomodulatory therapy, our findings suggest that pro-inflammatory programming of innate immune cells may still occur through mechanisms independent of systemic inflammatory signals. In particular, our *in vitro* experiments demonstrated that exposure to GCDCA without any additional pro-inflammatory stimulation such as TNF-α or LPS was sufficient to alter monocyte-derived macrophage differentiation. These results highlight the potential for bile acids to contribute to chronic inflammation in PSC, even in the presence of immunosuppressive treatment, and emphasize the importance of considering local metabolic and immune interactions in the pathogenesis of PSC. These findings suggest that circulating primary bile acids may influence the peripheral immune system, which, upon recruitment to sites of colonic inflammation, could potentiate tumorigenic processes in PSC-IBD.

Our findings suggest that GCDCA modulates monocyte phenotype through bile acid receptor signaling, likely involving Takeda G protein-coupled receptor 5 (TGR5) or Farnesoid X receptor (FXR). While TGR5 is typically associated with anti-inflammatory responses [42], our data suggest that chronic GCDCA exposure drives a non-canonical monocyte activation pattern that may reflect a dysregulated immune adaptation. In the context of PSC-IBD, where bile acid homeostasis is impaired, such signaling could contribute to persistent low-grade inflammation, immune exhaustion, and a microenvironment conducive to colorectal cancer development.

Targeting bile acid signaling pathways such as FXR and TGR5 represents a promising avenue in PSC and potentially in PSC-IBD. TGR5 agonists, including INT-767 and INT-777, have shown preclinical efficacy in liver disease models by modulating inflammation and fibrosis [43]. However, their specific application in PSC-IBD remains to be explored. FXR-targeted therapies have progressed further clinically. The non-steroidal FXR agonist cilofexor has shown beneficial effects in phase II trials in PSC, including reductions in cholestatic markers such as alkaline phosphatase (ALP) and fibrosis-associated indicators like TIMP-1 [44]. Aldafermin (NGM282), an engineered FGF19 analog designed to avoid pro-proliferative risks, improved fibrosis markers but did not significantly alter ALP levels [45]. Obeticholic acid (OCA), a steroidal FXR agonist approved for primary biliary cholangitis (PBC), has also been tested in PSC. In the AESOP phase II trial, OCA treatment over 24 weeks led to reductions in ALP among PSC patients, approximately half of whom also had controlled IBD and were on stable ursodeoxycholic acid therapy [46]. These findings support the feasibility of targeting bile acid receptors in PSC and suggest potential for application in PSC-IBD, though further studies are needed to evaluate efficacy and safety in this specific population.

While previous studies linked bile acid profiles and GCDCA to IBD and CRC risk, our study builds on this by identifying GCDCA as a key marker in PSC with potential immunomodulatory effects and a mechanistic role in monocyte polarization [11,38]. This study offers several strengths, including the novel identification of a modulatory effect of GCDCA on monocyte differentiation toward a pro-inflammatory profile. Additionally, the combination of serum proteomics and comprehensive bile acid analysis provided a detailed assessment of systemic inflammatory and metabolic profiles. The integration of *in vitro* experiments to support findings in human cohorts strengthens the mechanistic understanding of the observed effects. Furthermore, the use of patient colon tissue to relate serum inflammatory profiles to pro-tumor gene expression in the colon adds a clinically relevant dimension, suggesting a potential link between systemic immune dysregulation and tissue-level pathology. However, this study has some limitations. The small, country-specific cohort size and the lack of an external validation cohort limit the generalizability of the findings. Moreover, the study lacked a comparison group with non-PSC cholestatic diseases, such as PBC, limiting the ability to determine whether elevated GCDCA influence in host immune regulation is specific to PSC or also relevant in other cholestatic conditions. Although no confounding effects were found within each subgroup (PSC and IBD), we recognize the heterogeneity within the PSC group, which included patients with UC, CD, no IBD, and cirrhosis. Future studies with larger cohorts and stratified analyses of specific PSC subgroups will be essential to validate and further explore these findings.

In conclusion, our clinical and experimental data support the hypothesis that, in patients with PSC, even the modest accumulation of BAs, such as GCDCA, in peripheral blood has an impact on circulating immune cells. This may be clinically relevant in PSC patients because circulating monocytes are primed by pro-inflammatory factors, and, when recruited to the inflamed colon, they undergo a functional shift that contributes to the characteristic colon-specific inflammation seen in PSC.

**Clinical Perspectives**

- Primary sclerosing cholangitis (PSC) is a chronic cholestatic liver disease frequently associated with inflammatory bowel disease (IBD). The role of circulating bile acids in modulating immune responses in PSC remains unclear. This study investigates the impact of bile acids, particularly glycochenodeoxycholic acid (GCDCA), on peripheral immune differentiation and colonic inflammation.
- Patients with PSC exhibited a distinct pro-inflammatory immune signature, with increased serum levels of conjugated primary bile acids and up-regulated inflammatory markers in colonic tissue. GCDCA was found to modulate monocyte differentiation, promoting a pro-inflammatory phenotype, which was further reflected in elevated tumor-associated markers in the colon.
- These findings suggest that bile acid-mediated immune dysregulation plays a key role in PSC pathology, contributing to systemic inflammation and a pro-tumorigenic colonic microenvironment. Targeting bile acid signaling may represent a therapeutic approach for PSC and its associated complications.

## Supplementary material

Online supplementary tables

## Data Availability

All data will be made available upon request. Proteomic data are available at PRIDE repository (identifier PXD051896) [21].

## Open Access

This article has been published open access under our Subscribe to Open programme, made possible through the support of our subscribing institutions, learn more here: https://portlandpress.com/pages/open_access_options_and_prices#conditional

## Abbreviations

ALP: alkaline phosphatase
5-ASA: 5-aminosalicylic acid
CCL2: chemokine c-c motif ligand 2
CD: Crohn’s disease
COX2: cyclooxygenase-2
DCA: deoxycholic acid
DIA: data-independent acquisition mode
FDR: false discovery rate
FXR: Farnesoid X Receptor
GCDCA: glycochenodeoxycholic acid
IBD: inflammatory bowel disease
IFN-γ: interferon-gamma
IL-6: interleukin-6
IL-8: interleukin-8
IL-33: interleukin-33
IQR: interquartile range
MBR: Match-between-runs
OCA: Obeticholic acid
PBC: primary biliary cholangitis
PSC: primary sclerosing cholangitis
SEM: standard error of the mean
TGR5: Takeda G protein-coupled receptor 5
UC: ulcerative colitis
log2FC: log2 fold change
