# Supplementary material for: Primary bile acid shapes peripheral immunity in inflammatory bowel disease-associated primary sclerosing cholangitis
Source: Clin Sci (Lond). 2025 Jun 23;139(12):703–16. doi: 10.1042/CS20256078 (PMC12312387; doi:10.1042/CS20256078)
Supplement: Online supplementary tables [file CS-139-12-CS20256078-s001.docx]

**Supplementary data**

**Table S1. List of primers used for mRNA gene expression analysis**

| **Gene** | **Forward sequence** | **Reverse sequence** |
| --- | --- | --- |
| *CCL2* | ACTGAAGCTCGCACTCTCGC | AGCCTCTGCACTGAGATCTTC |
| *COX2* | CGGTGAAACTCTGGCTAGACAG | GCAAACCGTAGATGCTCAGGGA |
| *FGF-19* | CGGAGGAAGACTGTGCTTTCG | CTCGGATCGGTACACATTGTAG |
| *FXR* | CTCATTGAACATTCCCATTTACCTAC | GGACCTGCCACTTGTTCTGTTA |
| *IL-8* | CTTGGCAGCCTTCCTGATTT | TTCTTTAGCACTCCTTGGCAAAA |
| *LGR5* | CTTCCAACCTCAGCGTCTTC | TTTCCCGCAAGACGTAACTC |
| *MUC2* | GAGGGCAGAACCCGAAACC | GGCGAAGTTGTAGTCGCAGAG |
| *NLRP3* | GGCAACACTCTCGGAGACAA | GGAAAGATCCCAGCAGCAGT |
| *SNAIL* | ACCACTATGCCGCGCTCTT | GGTCGTAGGGCTGCTGGAA |
| *TGR5* | AGACACCATGCACTTGGTCC | GTCCTTCCTGGGAGATGGCT |
| *TLR4* | CCCTGAGGCATTTAGGCAGCTA | AGGTAGAGAGGTGGCTTAGGCT |
| *TNF-α* | GACAAGCCTGTAGCCCATGTTGTA | CAGCCTTGGCCCTTGAAGA |
| *TWIST1* | GCCAGGTACATCGACTTCCTCT | TCCATCCTCCAGACCGAGAAGG |
| *β-ACTIN* | CTGGAACGGTGAAGGTGACA | AAGGGACTTCCTGTAACAACGCA |

**Table 2.** Serum string analysis of pathways related to immune response increased in patients with PSC-IBD compared with IBD alone

| Term ID | Term description | Strength | FDR | Matching proteins in your network (labels) |
| --- | --- | --- | --- | --- |
| GO:0140375 | Immune receptor activity | 0.78 | 0.0209 | CTSH,OSMR,LEPR,IL1R2,PIGR,CR2,IL6ST |
| hsa04151 | PI3K-Akt signaling pathway | 0.5 | 0.0230 | PCK2,OSMR,ITGA1,CSF1R,HSP90B1,MET,COL6A1,ITGB1,RELN |
| GO:0098742 | Cell-cell adhesion via plasma-membrane adhesion molecules | 0.81 | 2.31e-05 | CEACAM1,ICAM1,CDH6,CDH2,HMCN1,VCAM1,ALCAM,CADM1,SELE,PLXNB2,ITGB1,PVR,ROBO1,CDHR2 |
| GO:0002526 | Acute inflammatory response | 1.09 | 0.00015 | VCAM1,CD163,F8,VNN1,ASS1,B4GALT1,PRCP,SAA2 |
| GO:0042311 | Vasodilation | 1.19 | 0.00081 | APOE,ITGA1,EXT2,CPS1,SOD2,KNG1 |
| GO:0006954 | Inflammatory response | 0.54 | 0.0047 | SEMA7A,CSF1R,VCAM1,SELE,CD163,F8,VNN1,ASS1,B4GALT1,PRCP,ITGB1,GGT5,CD14,SAA2,KNG1 |
| GO:0007159 | Leukocyte cell-cell adhesion | 1.04 | 0.0112 | NT5E,ICAM1,VCAM1,SELE,ITGB1 |
| GO:0002376 | Immune system process | 0.27 | 0.0299 | CTSH,GPLD1,CTSD,ENPP2,USP14,SEMA7A,CD81,ICAM1,ITGA1,CSF1R,VCAM1,MERTK,ALCAM,FCAMR,C7,CADM1,LEPR,IL1R2,SELE,PIGR,DPP4,CR2,VNN1,CTSS,ASS1,PSMB8,B4GALT1,MFAP4,ITGB1,CD14,SOD2,KNG1 |
| GO:0006953 | Acute-phase response | 1.07 | 0.0324 | CD163,F8,ASS1,SAA2 |
| HSA-6798695 | Neutrophil degranulation | 0.73 | 4.47e-07 | CEACAM1,PYGL,PGRMC1,CTSH,CTSD,PSMB1,ANPEP,ACAA1,CTSB,PIGR,QSOX1,VNN1,CTSS,PGM1,B4GALT1,PRCP,ADA2,CD14,IDH1,LAMP2,MME |
| HSA-168249 | Innate immune system | 0.5 | 5.25e-05 | CEACAM1,PYGL,PGRMC1,CTSH,CTSD,USP14,PSMB1,CD81,HSP90B1,ANPEP,C7,ACAA1,CTSB,PIGR,CR2,QSOX1,VNN1,CTSS,PGM1,PSMB8,B4GALT1,PRCP,ADA2,CD14,IDH1,LAMP2,MME |
| HSA-168256 | Immune system | 0.38 | 6.21e-05 | CEACAM1,PYGL,PGRMC1,CTSH,CTSD,CANX,USP14,PSMB1,CD81,ICAM1,OSMR,CSF1R,VCAM1,HSP90B1,ANPEP,COL3A1,C7,IL1R2,ACAA1,CTSB,PIGR,CR2,QSOX1,VNN1,CTSS,GSTO1,PGM1,PSMB8,B4GALT1,IL6ST,PRCP,ITGB1,ADA2,CD14,IDH1,PVR,LAMP2,MME,SOD2 |
| HSA-449147 | Signaling by interleukins | 0.58 | 0.0030 | CANX,USP14,PSMB1,ICAM1,OSMR,CSF1R,VCAM1,HSP90B1,IL1R2,GSTO1,PSMB8,IL6ST,ITGB1,SOD2 |
| HSA-198933 | Immunoregulatory interactions between a lymphoid and a non-lymphoid cell | 0.75 | 0.0439 | CD81,ICAM1,VCAM1,COL3A1,ITGB1,PVR |
